# ArGD: An Integrated Database and Analysis Platform for *Artocarpus* Genomics and Transcriptomics

**DOI:** 10.3390/genes17010091

**Published:** 2026-01-16

**Authors:** Peng Sun, Hongyuan Xi, Lei Yang, Lianfu Chen, Ying Bao

**Affiliations:** 1 School of Life Sciences, Qufu Normal University, Qufu 273165, China; helloxi2004@126.com; 2 School of Computer Science and Technology, Shandong Jianzhu University, Jinan 250101, China; yanglei@sdjzu.edu.cn; 3 Plant Science and Technology College, Huazhong Agriculture University, Wuhan 430070, China; chenlianfu@mail.hzau.edu.cn

**Keywords:** *Artocarpus*, jackfruit, genome database, comparative genomics, genome synteny, functional genomics, secondary metabolism

## Abstract

Background:The genus *Artocarpus* includes about 70 species, such as the economically important jackfruit and breadfruit, which serve as vital sources of food, timber, and medicine in the tropics. However, systematic research and genetic improvement have been restricted by the scarcity and fragmentation of available genomic data. Methods: Here, we developed the *Artocarpus* Genome Database (ArGD), a publicly accessible, comprehensive research platform dedicated to this genus. ArGD centrally integrates high-quality genomic sequences from seven *Artocarpus* genomes, along with related transcriptomic data and detailed functional annotations. Results: Beyond basic data retrieval, ArGD features a suite of advanced visualization and analysis modules, including BLAST, JBrowse, expression heatmaps, volcano plots, synteny viewers, *Artocarpus*CYC metabolic interface, and Gene Ontology (GO)/KEGG enrichment analyses. Additionally, ArGD provides online identification tools for gene families related to fruit aroma and secondary metabolism. Conclusions: Overall, ArGD serves as a valuable resource for functional genomics and comparative studies in *Artocarpus*, facilitating future research and data-driven studies of genetic improvement.

## 1. Introduction

The genus *Artocarpus* (Moraceae) comprises approximately 70 tropical species [1], renowned for nutritious aggregate fruits and widely used as food, timber, and traditional medicine in their native regions [2]. This includes representative cultivated species, such as jackfruit (*Artocarpus heterophyllus* Lam.) and breadfruit (*Artocarpus altilis* (Parkinson) Fosberg), which have been domesticated for thousands of years and are distributed throughout tropical regions [3]. *Artocarpus* plants are typically rich in carbohydrates, vitamins, and other essential nutrients, holding potential for ensuring food security in tropical regions [4]. Their high-quality timber and diverse bioactive compounds are also highly valued [5]. In summary, they hold extensive value for biological and pharmaceutical research [6,7].

*Artocarpus* aroma represents an important target for breeding improvement. The characteristic fragrance comes from volatile esters, alcohols, aldehydes, and terpenoids working together [8]. Studies with gas chromatography-olfactometry (GC-O) and aroma extract dilution analysis (AEDA) showed that ethyl 3-methylbutyrate, ethyl butyrate, and 3-methylbutanal isomers are particularly important for aroma [9]. These volatile esters form through fatty acid and amino acid metabolism when specific enzymes act on alcohol precursors. Two important enzyme families in this process are the alcohol dehydrogenase (ADH) family, which converts aldehydes to alcohols, and the BAHD acyltransferase superfamily. Acyl-transferases catalyze the final esterification step, making them essential targets for understanding and improving fruit flavor [10]. Terpenoids represent the main secondary metabolites in plants and play important roles in *Artocarpus* fragrance, defense, and bioactivity [11,12]. Terpenoids are complex compounds composed of isoprene units, which can be categorized as mono-, sesqui-, di-, or triterpenes [13,14]. Terpenes are found throughout *Artocarpus* tissues—wood, bark, and leaves—in species like *A. altilis*, *Artocarpus lakoocha*, and *Artocarpus camansi* Blanco [15,16]. One example is limonene, an aromatic monoterpene identified in *Artocarpus* plants [17]. The terpene synthase (TPS) gene family is therefore worth investigating to understand these compounds in *Artocarpus* better.

While *Artocarpus* represents both an economically valuable and ecologically important genus, it has not received the genomic attention given to model plant systems [18]. Most molecular investigations have relied on Simple Sequence Repeat (SSR) analysis and transcriptome sequencing [19]. Prior to 2019, the field operated without a high-quality reference genome for any *Artocarpus* species. Only low-coverage genomic data from *A. camansi* existed, providing limited genomic context [19]. These constraints have limited progress in understanding the genetic basis of important agronomic and biochemical traits.

In recent years, advances in sequencing technology have enabled the decoding of genomes for several species within the genus *Artocarpus*. The African Orphan Crops Initiative completed draft genomes for jackfruit (*A. heterophyllus*, 0.98 Gb) and breadfruit (*A. altilis*, 0.83 Gb) [3]. Subsequently, high-quality assemblies emerged, including the chromosome-level genome of the endangered species *A. nanchuanensis* [20]. Based on literature searches, at least seven genomes from five *Artocarpus* species have been sequenced, assembled, and published to date [21]. However, genomic data remain scattered across publications and databases, lacking a unified query platform [3].

While numerous specialized genomic databases such as the Genome Database for Allium (AlliumDB, https://allium.qau.edu.cn, accessed on 1 December 2025), CottonGen (https://www.cottongen.org/, accessed on 1 December 2025), and a multi-omics database of M. sativa (MODMS, https://modms.lzu.edu.cn/, accessed on 1 December 2025) have recently emerged [22,23,24], comprehensive, publicly accessible genomic resource for the genus *Artocarpus* has been notably absent. To address this gap, we developed the *Artocarpus* Genome Database (ArGD; http://jack.ficusgd.com/Artocarpus, accessed on 5 December 2025), the first publicly accessible platform designed to integrate genomic data for this genus centrally. ArGD houses sequences and functional annotations from seven genomes of five *Artocarpus* species, alongside analytical tools including BLAST, JBrowse (v1.16), expression heatmaps, volcano plots, synteny viewers, and enrichment analysis. Additionally, the database features a “Family” module for identifying genes associated with aroma. By consolidating dispersed data into a unified, user-friendly platform, we anticipate ArGD will advance comparative genomics and trait gene mining, thereby directly supporting crop breeding and conservation efforts.

## 2. Materials and Methods

### 2.1. Genome Data Collection and Annotation

**Figure 1:**
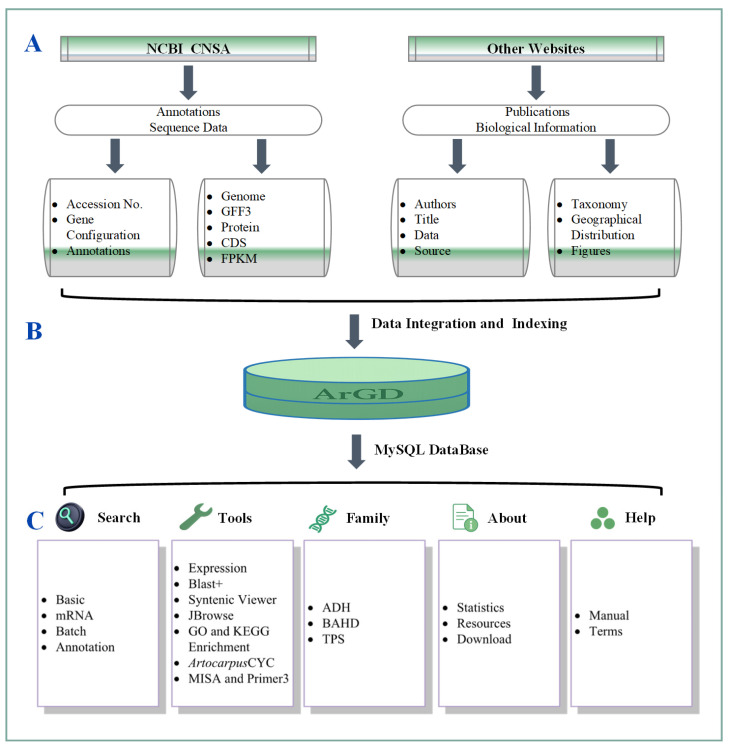
Overall architecture and functional framework of the ArGD. (**A**) Data collection from NCBI CNSA and other public resources, including genome assemblies, annotations, expression data, and biological information. (**B**) Standardization, integration, and indexing of all datasets into ArGD implemented with a MySQL database. (**C**) User-oriented web modules for data search, genome visualization, bioinformatics analyses, and data access.

We collected seven *Artocarpus* genomes representing five species from various genomic repositories (Table 1 and Table 2, Figure 1A). *A. heterophyllus* S10 (NCBI accession: PRJNA788174), *A. camansi* (PRJNA301299), *A. nanchuanensis* (PRJNA624965), and *A. hirsutus* (PRJEB55580) were retrieved from the NCBI GenBank database [19,20,21,25]. The *A. heterophyllus* BARI_K3 assembly was acquired from the Global Institute for Food Security Biodata Portal (https://bdp.dma.gifs.ca/dataset/jackfruit-barc-gifs, accessed on 5 December 2025) [26]. Additionally, *A. altilis* and *A. heterophyllus* genomes were sourced from the AOCC ORCAE platform (https://bioinformatics.psb.ugent.be/orcae/aocc, accessed on 25 November 2025) [3]. All genomic datasets, including GFF3 annotation files, coding sequences (CDS), and proteomes, underwent standardized formatting verification and systematic organization using In-house Perl scripts to ensure compatibility with downstream annotation pipelines and integration into ArGD.

In the ArGD project, the assembled protein sequences were searched and annotated. This process involved aligning the sequences with the NCBI non-redundant protein database (Nr, v2024-10) using BLASTP (BLAST+ v2.13.0), with a cutoff E value of 10–5 [27]. Subsequently, the protein sequences were compared with a variety of other databases for function annotation, including Swiss-Prot (v2024-09), eggNOG (v6.0), Pfam (v2024-11), KEGG (v64.0), KOG (v2003-03), and InterPro (v5.72-103.0). It is worth noting that the InterPro annotation integrates the results of its eight member databases: CDD, FunFam, Gene3D, PANTHER, Pfam, PRINTS, SMART, and SUPERFAMILY [28]. To derive Gene Ontology (GO) terms related to molecular functions, cellular components, and biological processes based on Nr annotations, we used Blast2GO (https://www.blast2go.com/, accessed on 25 November 2025) for prediction [29]. The final result of this workflow was the generation of eight major functional annotation data categories for ArGD: Nr, Swiss-Prot, InterPro, Pfam, KOG, eggNOG, GO, and KEGG (Table 1).

### 2.2. RNA-Seq Data Processing

Raw paired-end RNA sequencing data for multiple *Artocarpus* species were acquired from the NCBI Sequence Read Archive (SRA, https://www.ncbi.nlm.nih.gov/sra/, accessed on 20 December 2025) and the China National Gene Bank Database (CNGBdb, https://db.cngb.org/, accessed on 24 November 2025).

Initial read quality was evaluated using FastQC (v0.12.1) with default parameters [30]. Adapters and low-quality sequences were subsequently removed using Trimmomatic (v0.30) [31] with the parameters ILLUMINACLIP:TruSeq3-PE-2.fa: 2:30:10 LEADING:5 TRAILING:5 SLIDINGWINDOW:4:15 MINLEN:60. The resulting high-quality reads were aligned to the corresponding *Artocarpus* reference genome employing Hisat2 (v2.2.1) [32] with the following parameters: --dta --very-sensitive. Transcript assembly and expression quantification were conducted using StringTie (v2.2.1) [33], applying parameters -m 200 -f 0.3; individual sample assemblies were subsequently merged using StringTie’s merge function to create a unified, non-redundant transcript set. Gene expression levels were normalized and reported as Transcripts Per Million (TPM) or Fragments Per Kilo base of transcript per Million mapped reads (FPKM), calculated via the Ballgown R package in R (v4.3.2) [34]. Furthermore, we obtained the mean and standard error of the TPM/FPKM values for biological replicates (Figure S1). Finally, a comprehensive expression matrix was generated after TMM (Trimmed Mean of M-values) normalization [35].

The expression matrix was transformed using log_2_(TPM/FPKM+1) to generate online heatmaps in the “Expression” module. Additionally, we integrated the BAM files from the alignment step into JBrowse for visualization. For datasets containing at least three biological replicates, differential expression analysis was conducted using DESeq2 and edgeR in R based on raw gene-level read count matrices derived from the alignment (BAM) files using the reference gene annotation.

### 2.3. Comparative Genomics Analysis

For synteny analysis, a subset of five ArGD genomes was curated based on scaffold N50 continuity. We conducted pairwise protein comparisons using DIAMOND v2.1.1 [36] under stringent constraints: an E-value threshold of 1 × 10^−5^ and a maximum retention of five target sequences per query (--max_target_seqs 50). After that, we used the MCScanX v1.0 [37] software to determine the collinear blocks based on BLASTP comparison results and gene positions under default parameters. A total of 17,727 collinear blocks and 425,439 homologous gene pairs were identified in the genomes of the five *Artocarpus* species (Table 1). There were 1700–2600 collinear blocks and 41,000–51,000 homologous genes between any two genomes in the selected five *Artocarpus* genomes.

### 2.4. Metabolic Pathway Prediction

The seven genomes in ArGD were subjected to metabolic pathway prediction using Pathway Tools software (v24.0) [38]. For each species, the analysis comprehensively considered both the genome and individual gene sets (*A. camansi* gene sets were deduplicated using MMseqs2 with default parameters) [39], alongside gene functional descriptions from the Enzyme Commission (EC) and AHRD (https://github.com/groupschoof/AHRD, accessed on 21 November 2025), as well as relevant data from the SwissProt database. We consolidated all this information into a single PF-format file and used Pathway Tools’ Pathologic module to predict relevant pathways. Finally, the *Artocarpus*CYC online database was created using the Pathway Tools (v24.0) web server. This database represents a novel pathway/genome database (PGDB) containing predicted metabolic pathways for organisms, enabling users to browse, search, and perform comparative analyses based on predicted pathways [40].

### 2.5. Database Architecture and Implementation

The ArGD Database was architecturally designed and implemented using the established LAMP (Linux, Apache, MySQL, Perl/PHP v8.1) software stack, providing a robust and scalable foundation. The server operates on Rocky Linux (v9.2), with the Apache (v2.4.53) HTTP Server managing web requests and MySQL (v8.4.0) serving as the relational database management system for genomic data (Figure 1B). The frontend user interface was developed using standard web technologies (HTML, CSS, JavaScript) to ensure broad compatibility. To enhance user experience and interactivity, the Vue.js framework (https://vuejs.org/, accessed on 27 November 2025) was integrated for building responsive user interface components, and the Plotly.js library (https://plotly.com/, accessed on 25 November 2025) and D3.js (https://d3js.org, accessed on 25 November 2025) [41] library was employed for creating dynamic, interactive data visualizations directly within the browser. The entire system is hosted on dedicated hardware featuring an Intel Xeon E5-2630V4 CPU and 64 GB of RAM, ensuring sufficient resources for database operations.

The platform incorporates built-in tools for streamlined genomic research. Interactive exploration of the *Artocarpus* genome assembly, gene models, and other annotations is facilitated by an embedded instance of JBrowse [42]. SequenceServer v3.1.3 [43] was deployed to enable graphical BLAST+ searches against genomic, transcriptomic, and proteomic datasets. Additionally, a custom gene set enrichment analysis module (including GO and KEGG) was developed to enable functional interpretation of gene lists. This module utilizes the BioPerl toolkit for core bioinformatics tasks such as parsing gene identifiers and retrieving annotations [44], while the statistical analysis, including significance testing for functional category over-representation (e.g., GO terms), is performed using the R language and environment for statistical computing.

Furthermore, ArGD integrates ADH/BAHD/TPS identification tools using Bioperl modules to support research on *Artocarpus* secondary metabolism and breeding [45]. Data visualization (e.g., heatmaps, volcano plots) is powered by Plotly.js (v2.27), supplemented by embedded pipelines for synteny analysis, *Artocarpus*CYC, and MISAweb [46].

## 3. Results and Utility

### 3.1. Overview of ArGD

The ArGD features a streamlined structure with six main modules: Genome, Search, Tools, Family, Document, and Community (Figure 1C). The integrated “Tools Module” provides various bioinformatics applications for online genomic analysis, while the “Family Module” offers three aromatic-related gene family online identification toolkit, and the “About Module” covers statistics and download tools, users can freely download GFF3 annotations and sequence data (genome, CDS, protein) for all seven *Artocarpus* assemblies from ArGD, whereas the “Help Module” handles user manuals and copyright terms. This platform enables researchers to access, analyze, and visualize genomic data within the *Artocarpus* genus, supporting studies in comparative genomics and functional annotation.

### 3.2. Search and Homology Alignment

**Figure 2:**
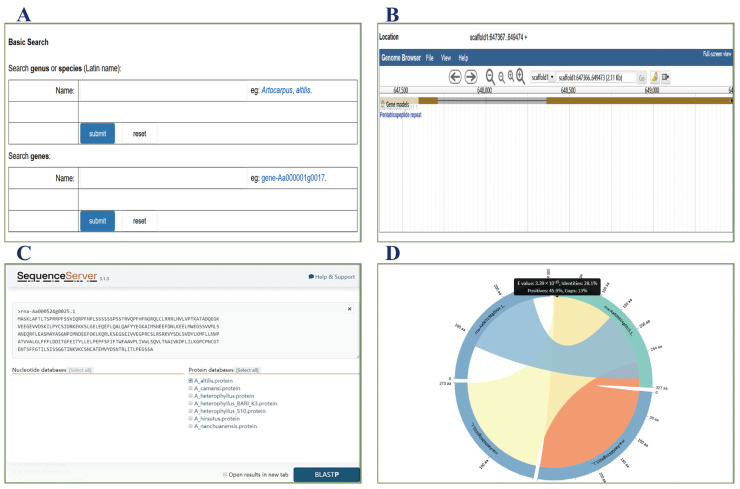
Search, Blast Search, GeneModel exhibition of the ArGD. (**A**) Basic search interface for species and gene queries. (**B**) Genome browser showing gene location and structure through Basic Search of (**A**). (**C**) SequenceServer BLASTP results against *Artocarpus* protein databases. (**D**) Species distribution of homologous sequences with E-value and identity statistics, the label ‘aa’ indicates amino acid positions.

The ArGD database provides comprehensive search functionalities enabling efficient retrieval of genomic information through multiple query approaches (Figure 2A). Users can search for individual genes by ID or name to access detailed genomic features through the integrated JBrowse genome browser, which facilitates precise localization and visualization of gene structures, sequences, and associated gene models (Figure 2B). Similarly, mRNA-based searches provide transcript-specific structural and sequence information with synchronized functional annotations from InterPro, KEGG, Pfam, and GO databases displayed on corresponding result pages.

Beyond fundamental searches, ArGD offers advanced batch processing and ontology-based querying. The “Batch Search” module enables simultaneous querying and downloading of mRNA and protein sequences for multiple genes. At the same time, the “Annotation Search” function facilitates bulk retrieval of functional annotations from Nr, SwissProt, KOG, and eggNOG databases. Additionally, users can perform ontology-based searches using specific terms such as GO/KEGG identifiers or InterPro domain classifications to identify genes associated with particular biological functions.

SequenceServer v3.1.2 is deployed in ArGD to enable homology search. SequenceServer can call Blast+ after ArGD to perform blastn and blastx homology comparisons, enabling homology comparisons across seven *Artocarpus* genomes and CDS and protein sequence libraries. Users can select different databases on the page and select corresponding algorithms for homology comparison. At the same time, users can also customize e-value and the maximum number of matches (Figure 2C). SequenceServer provides comparison results in three different formats for download: FASTA, XML, and TSV. Moreover, users can synchronize comparison results online. SequenceServer also displays the comparison results in a circular diagram and lists the sequence numbers in the comparison in order by score, which is convenient for users to browse and view (Figure 2D).

### 3.3. Gene Expression Visualization

**Figure 3:**
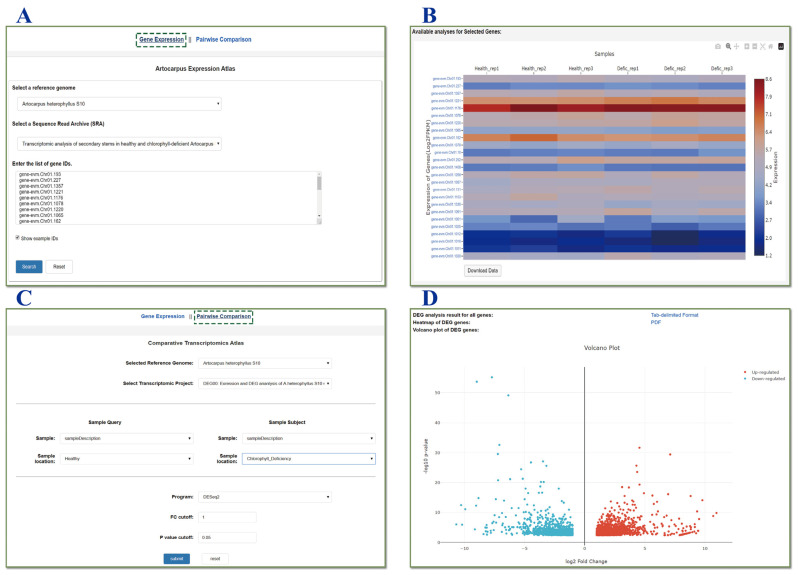
Representative screenshots for the Expression module. (**A**) Search interface of the “*Artocarpus* Expression” atlas, allowing users to select a reference genome, an SRA project, and input gene IDs. (**B**) Heatmap visualization displaying the expression profiles of the queried genes. (**C**) Parameter setting interface for “Pairwise Comparison” in the Comparative Transcriptomics Atlas to identify differentially expressed genes. (**D**) Volcano plot visualizing the differentially expressed genes (DEGs) resulting from the pairwise comparison.

To facilitate the analysis of RNA sequencing data, including pinpointing gene expression trends, ArGD does more than statically store RNA sequencing data and gene expression datasets; it introduces the “Gene Expression” module for dynamically displaying expression heatmaps of selected genes and pinpointing gene expression trends. The module provides downloadable file functionality, containing a complete list of identified genes and their associated details. Clicking on the gene ID in the first column of the heatmap takes the user to a page of gene structure and functional features. The “Gene Expression” module uses a heatmap tool powered by Plotly’s JavaScript library (https://plot.ly, accessed on 25 November 2025) to graphically present the expression profile of a selected genome, and the module enhances user engagement through interactive visualization tools. The basic principles of the “Gene Expression” module presentation are: (1) All expression data quantified in log_2_(TPM/FPKM+1) values are pre-imported into the MySQL database, (2) Interactive dynamic charts and graphs that can be integrated into web pages are created using the Plotly library for JavaScript (Figure 3A,B).

In addition to the “Gene Expression” module, a “Pairwise Comparison” module supports differential expression analysis for datasets containing three or more biological replicates. Users select query and test samples, as well as the analysis method (DESeq2 or edgeR). ArGD then performs the analysis and provides a downloadable differentially expressed gene (DEG) table (containing log_2_fold change values and P-values), an interactive volcano plot for online visualization, and an interactive heatmap for expression pattern analysis (Figure 3C,D).

### 3.4. Genome Browser and Synteny Viewer

**Figure 4:**
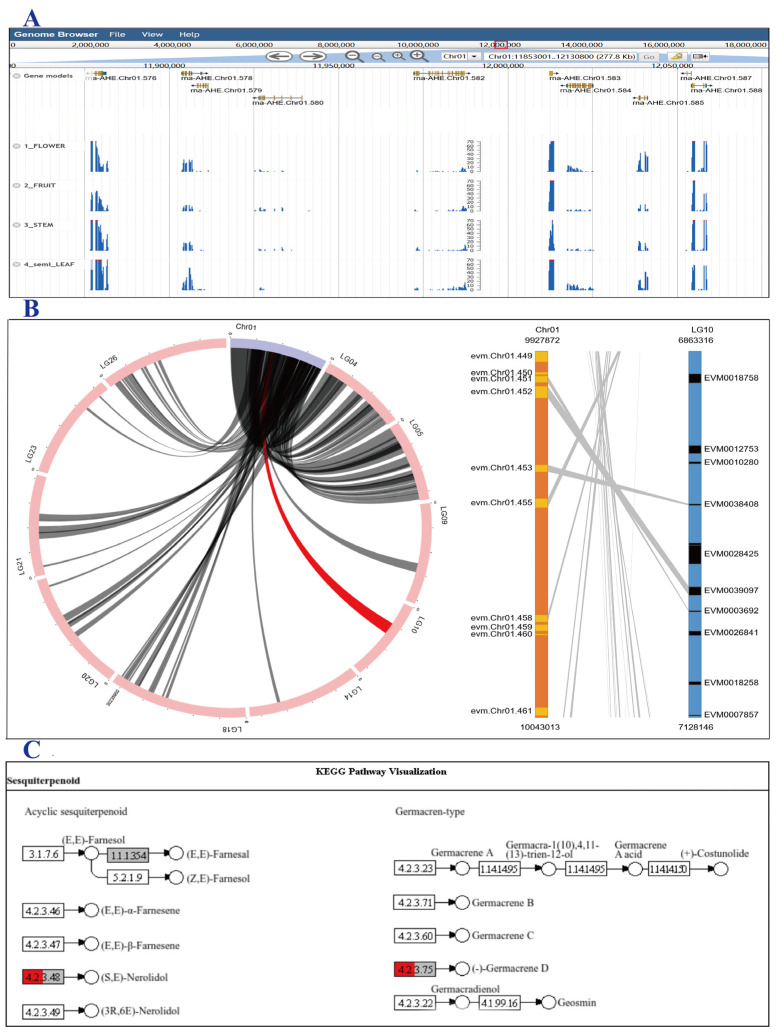
JBrowse genomic visualization, synteny viewer, and enrichment functions in ArGD. (**A**) JBrowse displaying gene models and tissue-specific RNA-seq expression profiles. (**B**) Circular synteny plot and detailed gene orthologous relationships. (**C**) KEGG pathway enrichment visualization of the sesquiterpenoid and triterpenoid biosynthesis pathway (ko00909), with enriched genes highlighted in red.

We have integrated the JBrowse genome browser on the ArGD website. The genome and structural sequence information of the seven *Artocarpus* species collected by ArGD have been imported into JBrowse. Users can browse the detailed genome, gene, mRNA, CDS, exon, intron, and other structural and sequence information of each species through JBrowse. At the same time, gene expression information is integrated into the JBrowse browser via BAM files, and users can browse expression peak graphs online for comparison and viewing (Figure 4A).

In ArGD, we integrated the “Synteny Viewer” function module, which can display the circle diagram of collinearity between species and the linear synteny plot of each collinear block through D3.js. We imported the collinearity information between different species into the backend MySQL database of the “Synteny Viewer” module. On the frontend collinearity selection page, users can manually select one or more chromosomes or scaffolds from their genome, and then select another species to be compared. The “Synteny Viewer” module can pass this information to the backend and generate a collinear Circos plot. Clicking each collinear block on the Circos plots can display detailed information about homologous genes. After clicking, the “Synteny Viewer” module displays a linear synteny plot on another web page, which contains information about homologous gene pairs, and can be zoomed in or out using the mouse wheel. Each gene name in the bar chart is clickable, allowing users to explore and browse detailed information for each gene. Therefore, in addition to using graphical, intuitive displays such as Circos plots and block information diagrams, the “Synteny Viewer” module can also browse the chromosome information for each block and various structural and functional information for each gene online. Consequently, an integrated platform for collinearity display and data querying was established (Figure 4B).

### 3.5. Enrichment Analysis

Enrichment analysis includes “GO Enrichment” and “KEGG Enrichment”, which can interpret and accurately locate specific gene clusters or families in biological datasets that appear more frequently than expected. Interpreting these gene combinations is crucial to grasping the regulatory dynamics of key biological functions and metabolic pathways, thereby mining and discovering genes of interest to other researchers. Based on this, ArGD integrates two functional modules, “GO Enrichment” and “KEGG Enrichment”. These two functional modules can receive gene sequence sets from frontend users and use all gene sets from the entire species as background to perform enrichment analysis using hypergeometric tests, thereby identifying significantly enriched GO terms and metabolic pathways. In addition, the enrichment analysis module generates high-quality visualizations, utilizing ggplot2 for bubble charts and pathview for KEGG pathway mapping (Figure 4C).

### 3.6. Aroma-Related Gene Families

Both the ADH and BAHD gene families are related to the synthesis of volatile esters, the main components of jackfruit aroma. In ArGD, we have integrated online identification tools for these two gene families. Users can identify gene families using local and imported protein sequences via file or text input. The tool, running on a server backend, can invoke HMMsearch (v3.3.1) to identify gene families based on the corresponding PFAM models. Users can also choose the ‘All’ or ‘Both’ PFAM/Motif buttons to select corresponding gene identification results; clicking on a gene ID then reveals detailed structural and functional information.

**Figure 5:**
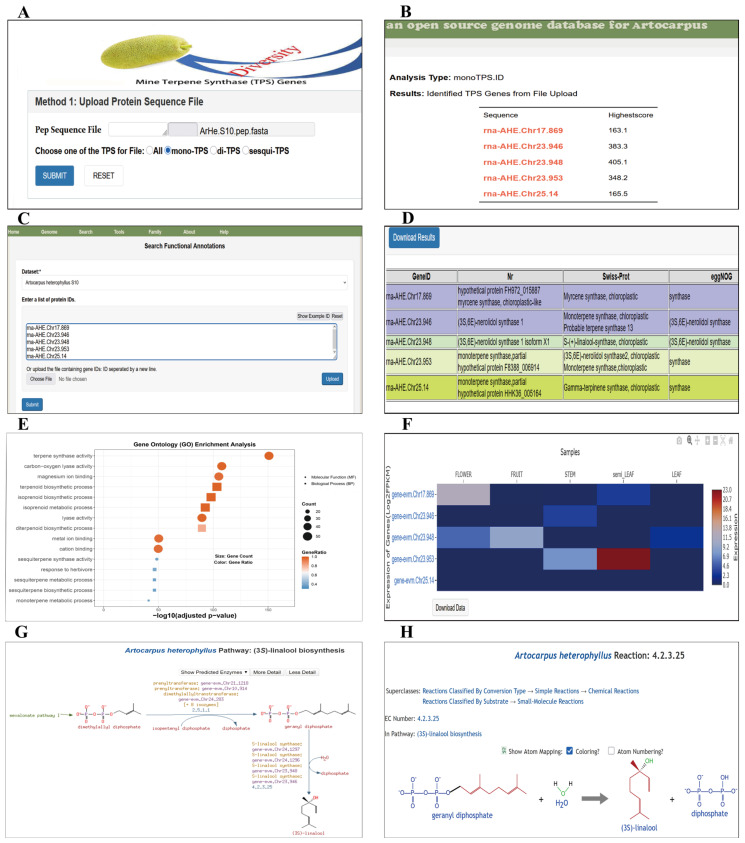
ArGD-based workflow for exploring putative mono-TPS candidates in *A. heterophyllus* S10. (**A**) “TPS Family” module showing protein input and TPS subfamily selection (mono-TPS). (**B**) Output list of ranked mono-TPS candidates from the mining step. (**C**) Functional “Annotation Search” interface for batch querying candidate IDs. (**D**) Integrated annotation summary from Nr, Swiss-Prot, and eggNOG. (**E**) “GO Enrichment” module analysis results for the candidate set (terpene-related terms). (**F**) “Expression” heatmap of candidate genes across available RNA-seq tissues/samples. (**G**) “*Artocarpus*CYC” module showing the view of the putative (3S)-linalool biosynthesis pathway with candidates mapped to pathway steps. (**H**) Reaction page for EC 4.2.3.25 showing the predicted substrate–product conversion in the pathway.

Traditional gene family identification methods can only identify a wide range of TPS genes, but cannot classify mono-, sesqui-, and diterpenes, etc. To accurately identify different types of TPS, ArGD has integrated an online TPS identification tool. This tool provides a web-based interface enabling users to submit protein sequences either through file upload or direct text input. Submissions are processed by a backend script that invokes a Perl program [45] to perform TPS identification and classification (https://github.com/liliane-sntn/TPS, accessed on 3 December 2025). The core analysis pipeline implements the search_TPS bioinformatics method, which utilizes HMMER searches against a curated database of TPS-specific profile Hidden Markov Models (HMMs) and PFAM models. This allows for the retrieval of high-confidence TPS candidates, which are further classified into putative mono-, sesqui-, or di-terpene synthases based on the best-matching profile HMM hits (Figure 5A,B).

### 3.7. Integrated Analysis Tools: MISA, Primer3, and ArtocarpusCYC

To support genetic marker discovery, ArGD provides a built-in MISA-web tool. Users can upload or paste sequences to search for microsatellites (SSRs) within their genomic data [46]. This tool rapidly processes input data and delivers comprehensive analysis results, including detailed statistics and a complete list of all SSRs. Additionally, we provide a standalone implementation of Primer3 (v4.1.0) for comprehensive primer design across diverse sequence contexts and experimental requirements. These functionalities support molecular marker development and genetic diversity analyses in jackfruit research.

The *Artocarpus*CYC sub-website displays genomic information in multiple formats, including genome sequences, metabolic pathways, and compound data. Using Pathway Tools annotation data, it presents the complex metabolic pathways from seven *Artocarpus* genome assemblies.

### 3.8. Case Study: A Workflow for Analyzing Monoterpene Synthase Genes Using ArGD

As a test case, we looked at TPS genes from the *A. heterophyllus* S10 genome. Using ArGD’s “TPS Family” module, we screened all predicted protein sequences for TPS candidates. With the ‘All’ option (Figure 5A), a total of 50 TPS genes were identified at the genome-wide level, outlining the overall size of the TPS family in this species. When we use the ‘mono-TPS’ option (Figure 5A), five putative monoterpene synthase candidates were selected based on their conserved domain features and internal scoring (Figure 5B). We then used the functional “Annotation Search” module to check these candidates against multiple databases, including Nr, Swiss-Prot, and eggNOG (Figure 5C). The annotation results were consistent with their classification as monoterpene synthase (Figure 5D). “GO Enrichment” module analysis showed enrichment in terpenoid/isoprenoid biosynthesis and metabolism, including monoterpene metabolic process, and molecular functions such as terpene synthase activity (Figure 5E).

To understand where and when these genes might function, we looked at their expression patterns using the transcriptomic data available in ArGD. Here, the input identifiers are linked to the corresponding gene entries for expression profiling. The heatmap showed clear differences across flower, fruit, stem, and leaf samples, which helped us decide which candidates to focus on (Figure 5F). We then examined the mono-TPS candidates in *Artocarpus*CYC at the pathway level, where a (3S)-linalool biosynthesis pathway was displayed, and several candidates were mapped to specific reaction steps (Figure 5G). For the final reaction, the EC 4.2.3.25 page shows how geranyl diphosphate (GPP) is converted to (3S)-linalool, making it easy to understand the predicted enzyme function (Figure 5H). These mono-TPS candidates also showed high sequence similarity to annotated monoterpene synthases in ArGD, supporting their putative functions.

ArGD also includes other analysis tools such as synteny visualization and online SSR identification. These tools can be useful for studying population genetics, developing molecular markers, and supporting breeding programs. This case study shows how ArGD allows researchers to move from genome-wide gene family analysis to functional interpretation and practical applications using a single integrated platform.

## 4. Discussion and Future Perspectives

In genomics research, specialized online platforms for specific genus or species are emerging as valuable alternatives to large public databases like NCBI, which often contain limitations and errors. These targeted platforms provide more accurate information and enable both in-depth taxonomic research and cross-disciplinary analyses, significantly improving research efficiency [47]. Based on this, ArGD came into being. As the first public, comprehensive integrated platform focusing on the genus *Artocarpus*, ArGD covers multiple fields, including genomics and functional genomics, comparative genomics, transcriptome and gene family related information analysis, and implements new functional modules such as “KEGG Enrichment” and aroma-related “Family”. ArGD will definitely be applied to the genome and transcriptome research of *Artocarpus*, and provide assistance for research in various fields such as improving the quality of Jackfruit fruit, disease resistance, molecular breeding, etc.

In the future, with the emergence of new *Artocarpus* genomes and other potential related genetic and multi-omics datasets, ArGD will be regularly updated and carefully organized to ensure the vitality and novelty of the ArGD database. Furthermore, we will continue expanding and updating ArGD with new functions, such as CMap gene linkage mapping [48], QTL identification, and CRISPR-Cas9 target prediction to enhance its analytical capabilities.

In summary, ArGD has created a new platform for genetics, evolutionary research, and biological applications of *Artocarpus* plants. As climate change, food security, and biodiversity conservation become increasingly urgent global challenges, ArGD will play a key role in the development of multifunctional applications and sustainable development research based on *Artocarpus*. We firmly believe that ArGD will develop into a comprehensive, globally accessible, and indispensable platform and become an important resource for users, plant breeders, and researchers in various fields who are committed to the genetic improvement of *Artocarpus*.

## Figures and Tables

**Table 1:** Summary of data types and contents currently available in ArGD.

Data Type	Entries No.	Details
Genome	7	Seven whole genome assemblies and annotations from five *Artocarpus* species *.
Species	5	Origin, genome groups, germplasm, sequences, and libraries, specific species pages with hyperlinks to various data and tools.
Gene	323,183	Genes from seven whole genome assemblies were parsed from NCBI nucleotide sequences.
mRNA	331,384	mRNAs form seven genome assemblies.
Protein	331,384	Proteins from seven genome assemblies.
Function annotation	8	Nr, SwissProt, KOG, and eggNOG annotations for seven *Artocarpus* genomes can be viewed through “Annotation Search” in ArGD; Pfam, InterPro (including CDD, FunFam, Gene3D, PANTHER, PRINTS, SMART, SUPERFAMILY), GO, and KEGG annotations can be viewed through “mRNA/Gene Search” on the ‘Gene Model’ page.
Transcriptomic datasets	7	RNA-seq datasets derived from five SRA projects (PRJNA311339, PRJNA791757, PRJNA1034797, PRJNA611876, PRJNA788174) and two CNSA projects (CNP0000715 and CNP0000486), including multiple condition-specific expression profiles.
Syntenic blocks	425,439	425,439 homologous gene pairs of five *Artocarpus* (*A. altilis*, *A. nanchuanensis*, *A. heterophyllus* ICRAFF11314, *A. heterophyllus* S10, *A. heterophyllus* BARI_K3) genomes.

* The five *Artocarpus* are designated as *A. altilis*, *A. heterophyllus*, *Artocarpus hirsutus*, *Artocarpus nanchuanensis*, and *A. camansi*.

**Table 2:** Genome assembly quality, RNA-seq, and annotation statistics of *Artocarpus* genomes included in ArGD.

Species	Genome Version	Assembly Size (Mb)	Ploidy	Scaffold N50 (Mb)	Busco V5 (%)	Gene No.	mRNA No.	Protein No.	RNA-Seq Projects
*A. altilis*	v1	833.04	2n = 2x = 28	1.54	95.2	34,010	34,010	34,010	CNP0000715, PRJNA311319, PRJNA791757
*A. heterophyllus*	v*ICRAFF_11314*	982.02	2n = 2x = 28	0.548	95.0	35,858	35,858	35,858	CNP0000486
*A. heterophyllus*	v*S10*	985.63	2n = 2x = 28	32.8	93.5	41,997	41,997	41,997	PRJNA788174, PRJNA611876
*A. heterophyllus*	v*BARI_K3*	843.00	2n = 2x = 28	0.425	97.2	41,083	48,685	48,685	/
*A. hirsutus*	v1	796.16	2n = 2x = 28	0.0499	96.7	46,137	46,137	46,137	/
*A.nanchuanensis*	v1	769.44	2n = 2x = 28	25.2	97.9	41,636	41,636	41,636	PRJNA1034797
*A. camansi*	v1	631.30	2n = 2x = 28	0.00243	96.6	82,462	83,061	83,061	/

## Data Availability

All data in ArGD are available at http://jack.ficusgd.com/Artocarpus freely (accessed on 1 December 2025). This database is hosted within the Moraceae Genome Database framework to centralize genomic resources for the Moraceae family. Scripts and workflows are publicly available via Zenodo (DOI: 10.5281/zenodo.18217353).

## Supplementary Materials

The following supporting information can be downloaded at https://www.mdpi.com/article/10.3390/genes17010091/s1, Figure S1. RNA-seq data processing, quantification, and differential expression workflow implemented in ArGD.; Table S1. Sensitivity analysis of DIAMOND parameter settings on synteny detection.
