# Supplementary material for: ArGD: An Integrated Database and Analysis Platform for Artocarpus Genomics and Transcriptomics
Source: Genes (Basel). 2026 Jan 16;17(1):91. doi: 10.3390/genes17010091 (PMC12840728; doi:10.3390/genes17010091)
Supplement: Supplementary file 1 [file genes-17-00091-s001.zip › genes-4080032-supplementary.pdf]

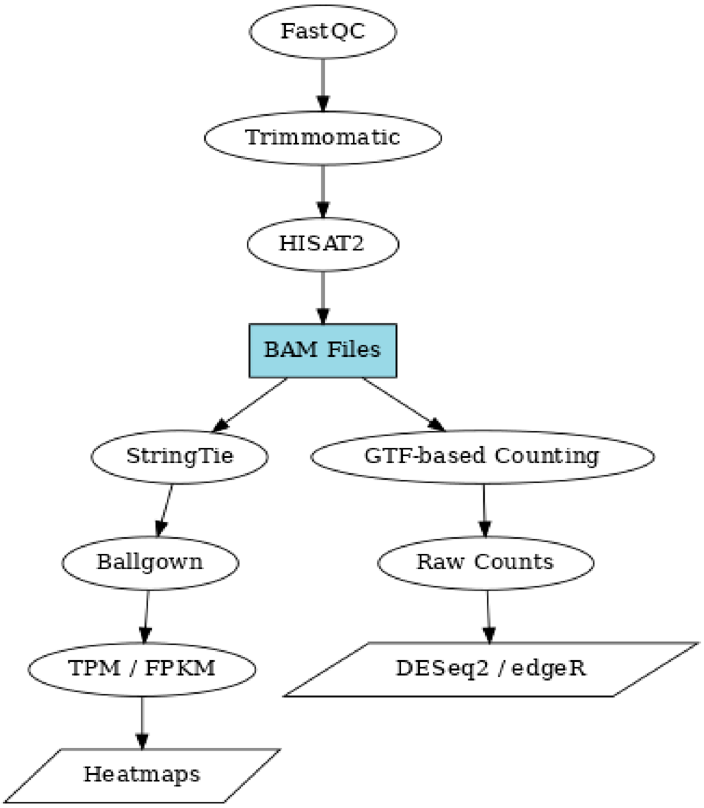

Figure S1. RNA-seq data processing, quantification, and differential expression workflow implemented in ArGD.

Table S1. Sensitivity analysis of DIAMOND parameter settings on synteny detection.

Parameter setting	DIAMOND max-target-seqs	Post-parse max hits	Reciprocal filtering	Total syntenic blocks	Total collinear gene pairs	Median genes per block	Mean genes per block
Stress test (low cap)	5	100	No	16,982	389,214	18	24.6
Medium cap	20	100	No	17,401	412,873	19	25.1
Default (used in study)	50	100	No	17,727	425,439	19	25.4
Reciprocal filtering	50	100	Yes	16,215	372,908	20	26.8
